# Data for characterisation of nanoformulations formed by cationic 1,4-dihydopyridine and calix[4]arene compositions

**DOI:** 10.1016/j.dib.2022.107988

**Published:** 2022-02-23

**Authors:** Martins Rucins, Roman Rodik, Aiva Plotniece, Nadiia Pikun, Mara Plotniece, Arkadij Sobolev, Vitaly Kalchenko, Karlis Pajuste

**Affiliations:** a Latvian Institute of Organic Synthesis, Aizkraukles str. 21, Riga LV-1006, Latvia; b Institute of Organic Chemistry, National Academy of Science of Ukraine, Murmanska str. 5, Kiev 02660, Ukraine; c Department of Pharmaceutical Chemistry, Faculty of Pharmacy, Riga Stradiņš University, Dzirciema str. 16, Riga LV-1007, Latvia

**Keywords:** Synthetic lipids, 1,4-dihydropyridine, Calix[4]arene, Nanoparticles, DLS

## Abstract

In this data file the characterisation of nanoformulations obtained from calix[4]arene/1,4-dihydropyridine (1,4-DHP) compositions in the various component ratio in an aqueous medium was performed by dynamic light scattering (DLS) technique. The hydrodynamic diameters of nanoparticle main population, polydispersity index and stability of nanoformulation were determined. In this article provided data are directly related to the previously published research articles – “Gene delivery agents possessing antiradical activity: Self-assembling cationic amphiphilic 1,4-dihydropyridine derivatives” [1], and “Studies of the physicochemical and structural properties of self-assembling cationic pyridine derivatives as gene delivery agents” [2] where was described synthesis, transfection activity of 1,1′-((3,5-bis((dodecyloxy)carbonyl)-4-phenyl-1,4-dihydropyridine-2,6-diyl)bis(methylene))bis(pyridin-1-ium) dibromide presented in this data file; and with articles “Cationic amphiphilic calixarenes to compact DNA into small nanoparticles for gene delivery” [3] and “Self-aggregation in aqueous solution of amphiphilic cationic calix[4]arenes. Potential use as vectors and nanocarriers” [4] where was described synthesis and ability to condense DNA for also mentioned calix[4]arenes – 5,11,17,23-tetra-(3-methylimidazolium)-methylene-25,26,27,28-etradodecyloxycalix[4]arene tetrachloride, 5,11,17,23-tetra(N,N-dimethyl-N-hydroxyethylammonium)-methylene-25,26,27,28-tetradodecyloxycalix[4]arene tetrachloride and 5,11,17,23-tetra(N,N-dimethyl-N-hydroxyethylammonium)-methylene-25,26,27,28-tetrahexadecyloxycalix[4]arene tetrachloride. Information provided in this data file can be used in medicinal chemistry for development of novel synthetic lipid nanoformulations.

## Specifications Table

**Table:** 

Subject	Organic Chemistry
Specific subject area	Organic synthesis and physicochemical characterisation of nanoformulations obtained from cationic amphiphilic 1,4-dihydopyridine and calix[4]arenes
Type of data	Table Figure
How the data were acquired	Dynamic light scattering (DLS) instrument Zetasizer Nano ZSP (Malvern Panalytical Ltd.) with Malvern Instruments Ltd. Software 7.12 was used for evaluation of self-assembling properties of pure cationic 1,4-DHP and calix[4]arene derivatives and characterisation of obtained nanoparticles formed by them in different ratios or individually. Scanning electron microscopy (SEM) has been used for nanostructure analysis (Field Emission Scanning Electron Microscope Hitachi S-4800, 10kV, 10 µA).
Data format	Raw and analysed
Description of data collection	The DLS measurements were carried out on a Zetasizer Nano ZSP (Malvern Panalytical Ltd., Malvern, UK) instrument with Malvern Instruments Ltd. Software 7.12, specifications - medium: water; refractive index: 1.33; viscosity: 0.8872 cP; temperature: 25 °C; dielectric constant: 78.5; nanoparticles: liposomes; refractive index of materials: 1.60; detection angle: 173°; wavelength: 633 nm. Field Emission Scanning Electron Microscope Hitachi S-4800, 10 kV, 10 µA was used for nanostructure analysis.
Data source location	Latvian Institute of Organic Synthesis, Riga, Latvia
Data accessibility	Repository name: Mendeley Data Data identification number: 10.17632/fnk8krrgpy.1 Direct URL to data: https://data.mendeley.com/datasets/fnk8krrgpy/1
Related research article	O. Petrichenko, M. Rucins, A. Vezane, I. Timofejeva, A. Sobolev, B. Cekavicus, K. Pajuste, M. Plotniece, M. Gosteva, T. Kozlovska, A. Plotniece, Studies of the physicochemical and structural properties of self-assembling cationic pyridine derivatives as gene delivery agents, Chem. Phys. Lipids, 191 (2015) 25-37. 10.1016/j.chemphyslip.2015.08.005. F.J. Ostos, J.A. Lebrón, P. López-Cornejo, M. López-López, M. García-Calderón, C.B. García-Calderón, I.V. Rosado, V.I. Kalchenko, R.V. Rodik, M.L. Moyá, Self-aggregation in aqueous solution of amphiphilic cationic calix[4]arenes. Potential use as vectors and nanocarriers, J. Mol. Liq., 304 (2020) 112724. 10.1016/j.molliq.2020.112724

## Value of the Data

• The data presented in this data file will contribute to the understanding of behaviour of nanocompositions comprised of cationic amphiphilic 1,4-dihydropyridine and cationic amphiphilic calix[4]arene which is based on dynamic light scattering measurements.
• This data will be useful for scientists working within the field of discovery and development of nanodelivery systems.
• The obtained data on properties of nanocompositions may serve as base for the design and development of new original delivery systems for incorporation of a gene/drug or diagnostic material for use in medicine.

## 1 Data Description

Lipid vesicles such as liposomes are particularly attractive as delivery systems for gene and drug delivery applications. Lipids are the basic components of the cell membrane and many lipids are highly biocompatible, omitting the problem of biodegradation [5]. A broad range of lipids is found in nature, while others can be obtained synthetically. The size and PDI values of liposomes are very important characteristic for drug delivery [6,7]. For drug delivery, the desirable size of liposomes usually ranges between 50 and 200 nm [8].

Previously our research groups reported data on cationic moiety containing 1,4-dihydropyridine (1,4-DHP) [1,9]. Cationic moieties containing calix[4]arene [3,10] derivatives synthesised as promising DNA delivery agents.

**Fig. 1:**
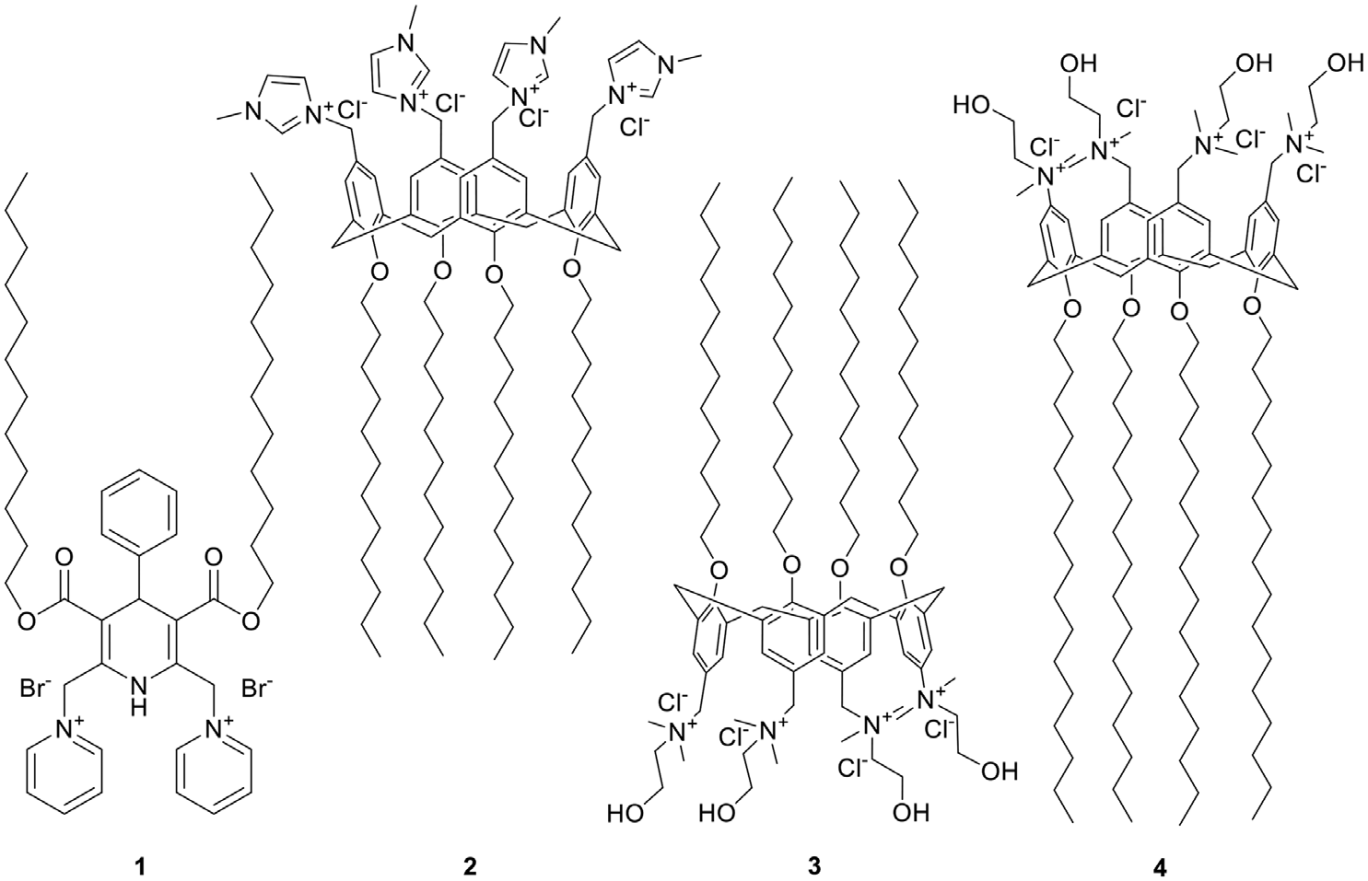
Structures of amphiphilic compounds 1–4 used for preparation of nanoformulations.

Now, we continue the studies on the original cationic synthetic lipid-like compounds, characterisation of formed nanoaggregates by them, elaboration of methods for preparation of composites and their characterisation. The structures of cationic amphiphilic 1,4-DHP and cationic amphiphilic calix[4]arene derivatives are depicted in Fig. 1.

The purities of the studied compounds were at least 97% according to high-performance liquid chromatography (HPLC) data.

Previously it was shown that both cationic amphiphilic 1,4-DHP derivatives [1,11] and cationic amphiphilic calix[4]arene derivatives [3,10] possessed self-assembling properties and formed liposomes, it was disclosed that they were capable to bound pDNA and transferred it into the cells.

In this data file, we provide characterisation of two-component nanoformulations comprised from cationic amphiphilic 1,4-DHP **1** as one component and the appropriate cationic amphiphilic calix[4]arene 2–4 as another component  at mol% ratios of 100/0, 75/25, 50/50, 25/75, and 0/100; starting from single the appropriate calix[4]arene (100/0) in the first sample and single 1,4-DHP (0/100) in the last sample. The obtained results - hydrodynamic diameters of the main population of nanoparticles (D), polydispersity index (PDI), zeta-potential (z-pot) and stability of nanoparticles formed by 1,4-DHP/calix[4]arene compositions in aqueous medium determined by the DLS method are presented in Table 1 (the graphs of nanoparticle size distributions (Figs. S1–S6) and zeta-potential (Figs. S7–S9) see in supplementary material; raw data in repository: Mendeley data 10.17632/fnk8krrgpy.1).

**Table 1:** The values hydrodynamic diameters of the main population of nanoparticles (D), polydispersity index (PDI), zeta-potential (z-pot) and stability of nanoparticles (fresly prepared and after storage of 20 days at rt) formed by calix[4]arene/1,4-DHP compositions in aqueous medium determined by the DLS method.

		Freshly prepared			After 20 days storage	
Compositions calix[4]arene/1,4-DHP	Ratio, mol%	D, nm	PDI	z-pot, mV	D, nm	PDI
**2**/**1**	100/0	159 ± 3	0.17 ± 0.02	51.8 ± 1.5	164 ± 1	0.15 ± 0.02
	75/25	163 ± 1	0.22 ± 0.02	56.0 ± 0.8	161 ± 3	0.19 ± 0.01
	50/50	287 ± 4	0.30 ± 0.04	52.6 ± 1.4	281 ± 3	0.28 ± 0.01
	25/75	159 ± 2	0.23 ± 0.02	55.9 ± 2.5	159 ± 1	0.21 ± 0.01
	0/100	115 ± 2	0.37 ± 0.01	53.7 ± 1.2	118 ± 2	0.39 ± 0.01

**3**/**1**	100/0	145 ± 48	0.55 ± 0.20	53.0 ± 1.5	159 ± 67	0.80 ± 0.30
	75/25	96 ± 7	0.77 ± 0.22	47.8 ± 2.0	79 ± 16	0.92 ± 0.10
	50/50	23 ± 1	0.51 ± 0.02	39.0 ± 1.5	24 ± 3	0.57 ± 0.16
	25/75	55 ± 1	0.45 ± 0.01	54.9 ± 4.3	57 ± 1	0.53 ± 0.01
	0/100	109 ± 1	0.29 ± 0.01	81.0 ± 6.4	109 ± 1	0.29 ± 0.01

**4**/**1**	100/0	155 ± 3	0.36 ± 0.03	59.5 ± 2.5	147 ± 2	0.27 ± 0.01
	75/25	157 ± 1	0.35 ± 0.01	52.6 ± 1.7	138 ± 1	0.25 ± 0.01
	50/50	138 ± 1	0.27 ± 0.01	63.3 ± 12.7	126 ± 1	0.24 ± 0.01
	25/75	154 ± 1	0.47 ± 0.02	49.1 ± 0.5	139 ± 2	0.30 ± 0.03
	0/100	103 ± 2	0.40 ± 0.01	58.3 ± 1.6	87 ± 1	0.35 ± 0.04

The obtained results demonstrated that for freshly prepared samples diameter of the main population of nanoparticles of single calix[4]arenes 2–4 was around 150 nm and for single 1,4-DHP was around 110 nm in all formulations. In drug delivery applications liposomes with PDI value equal or below 0.3 indicates an acceptable and homogenous nanoparticle population [12], whereas high PDI value is associated with a very broad size distribution (heterogeneity) [13]. Evaluation of PDI values of formulations demonstrated, that most homogenous samples were obtained for compositions of compounds **2**/**1** and **4/1**. PDI values of nanoformulation **2**/**1** were in the range 0.17-0.37 for freshly prepared samples and in the range 0.15-0.39 for samples after storage. PDI values of nanoformulation **4**/**1** were in the range 0.27–0.47 for freshly prepared samples and in the range 0.24–0.35 for samples after storage. While nanoformulation **3**/**1** was more heterogeneous system, which was confirmed by PDI values, giving values in the range 0.29-0.77 and 0.29-0.92 for freshly prepared samples and samples after storage, respectively. The diameter of the main population of nanoparticles for freshly prepared nanoformulation **2**/**1** were in the range 115-163 nm, with exception for ratio 50/50 where the diameter of the main population of nanoparticles was 287 nm, and the diameter of the main population of nanoparticles for nanoformulation **2**/**1** after storage was in the range 118–164 nm, with exception for ratio 50/50 where the diameter of the main population of nanoparticles was 281 nm. The diameter of the main population of nanoparticles for freshly prepared nanoformulation **4**/**1** was in the range 103–157 nm, and the diameter of the main population of nanoparticles for nanoformulation **4**/**1** after storage was in the range 87–147 nm. In the case of nanoformulation **3**/**1** the changes in values of diameter of the main population of nanoparticles were more pronounced depending on component ratio. For example, the value of diameter was 23 nm for composition 50/50 of the compounds, 55 and 96 nm for compositions 25/75 and 75/25 of compounds **3**/**1**, respectively. Similar data was observed also for this nanoformulation after storage for 20 days. Scanning electron microscopy (SEM) has been used for nanostructure analysis. The obtained images of freeze-dried samples of calix[4]arene **2**/1,4-DHP **1** compositions are provided in Supplementary material (Fig. S10).

Zeta-potentials for the tested samples were in the range of 39–81 mV which confirmed that the formed nanoparticle solutions were electrostatically stable. According to literature data z-pot over ± 20 mV verify stable nanoparticle solutions [14].

It was demonstrated that obtained nanoformulations were in the size range suitable for the development of the original delivery systems. Additionally, it was shown that the tested nanoformulations were stable for a period of 20 days which was confirmed by comparable nanoparticles physicochemical data – values of the diameter of the main population of nanoparticles and PDI values of the sample.

## 2 Experimental Design, Materials and Methods

Synthetic procedures for the preparation of these compounds 1–4 were described previously. Briefly, the elaborated synthesis of the cationic 1,4-DHP **1** involved three sequential steps. The first step was the synthesis of the parent 2,6-dimethyl 1,4-DHP derivative by a Hantzsch-type cyclisation using benzaldehyde, ammonium acetate and dodecyl acetoacetate. The second step involved the bromination of the methyl groups of the 2,6-dimethyl-1,4-DHP derivative with N-bromosuccinimide and the last step was the nucleophilic substitution of bromine at the 2,6-positions of-1,4-DHP by pyridine giving the target 1,1′-((3,5-bis((dodecyloxy)carbonyl)-4-phenyl-1,4-dihydropyridine-2,6-diyl)bis(methylene))bis(pyridin-1-ium) dibromide (**1**). More detailed synthetic procedures and characterisation of the original compounds were presented by Pajuste et al [15]. Cationic amphiphilic calix[4]arene derivatives 2-4 were synthesised through chloromethylation of parent calixarenes followed by substitution of chlorine with N-methylimidasole giving the target compound 5,11,17,23-tetra-(3-methylimidazolium)-methylene-25,26,27,28-etradodecyloxycalix[4]arene tetrachloride (**2**) or with N,N-dimethylethanolamine giving 5,11,17,23-tetra(N,N-dimethyl-N-hydroxyethylammonium)-methylene-25,26,27,28-tetradodecyloxycalix[4]arene tetrachloride (**3**) and 5,11,17,23-tetra(N,N-dimethyl-N-hydroxyethylammonium)-methylene-25,26,27,28-tetrahexadecyloxycalix[4]arene tetrachloride (**4**). More detailed synthetic procedures and characterisation of the original compounds were presented by Rodik et al [3,10].

### 2.1 Dynamic light scattering measurements

For the preparation of stock solution with concentration 10 mM an appropriate amount of compounds was dissolved in an appropriate amount of methanol. Due to a poor solubility, a stock solution of compound **4** was prepared at concentration 3.3 mM. Samples of compounds for dynamic light scattering (DLS) studies were prepared by thin-film hydration method in an aqueous solution at a final concentration of 0.5 mM for total lipid amount. The appropriate amount of stock solutions was used in order to obtain compositions of 1,4-DHP/calix[4]arene at stoichiometric mol% ratios of 100/0, 75/25, 50/50, 25/75, 0/100, after then the organic solvent was removed in vacuo and the residue was dried in high vacuo for 1 h. An appropriate amount of deionised water was added to each flask for the preparation of work solutions. Samples were prepared by sonication using a bath-type sonicator (Cole Parmer Ultrasonic Cleaner 8891CPX (Vernon Hills, IL, USA)). Samples were sonicated for 30 min at 50 °C.

The obtained aqueous solutions of nanoformulations were characterised by DLS measurements using a Zetasizer Nano ZSP (Malvern Panalytical Ltd., Malvern, UK) instrument with Malvern Instruments Ltd Software 7.12.; specifications– medium: water; refractive index: 1.33; viscosity: 0.8872 cP; temperature: 25 °C; dielectric constant: 78.5; nanoparticles: liposomes; refractive index of materials: 1.60; detection angle: 173°; wavelength: 633 nm. The data were analysed by using the multimodal number distribution software which was included with the instrument for this purpose. The measurements were performed in triplicate for each sample.

### 2.2 Scanning electron microscopy

The samples of compounds **2, 1** and their compositions after DLS measurements were freeze-dried. Each dry sample of obtained compositions was fixed on carbon adhesive tape folowed by sputter-coated with gold at thicknes of 3.0 nm. Scanning electron microscopy (SEM) has been used for nanostructure analysis (Field Emission Scanning Electron Microscope Hitachi S-4800, 10 kV, 10 µA).

### 2.3 Statistical analysis

Results are expressed as mean ±standard deviation (SD). All experiments were performed at least three times.

## Ethics Statements

Not applicable.

## CRediT authorship contribution statement

**Martins Rucins:** Conceptualization, Software. **Roman Rodik:** Supervision. **Aiva Plotniece:** Writing – original draft. **Nadiia Pikun:** Visualization, Investigation. **Mara Plotniece:** Data curation. **Arkadij Sobolev:** Writing – review & editing. **Vitaly Kalchenko:** Conceptualization, Methodology. **Karlis Pajuste:** Supervision.

## Declaration of Competing Interest

The authors declare that they have no known competing financial interests or personal relationships that could have appeared to influence the work reported in this paper.

## Data Availability

DLS-data for nanoparticle characterisation of 1,4-DHP and calix[4]arenes (Original data) (Mendeley Data).

## Appendix Supplementary materials

Image, application 1
