# Supplementary material for: Data for characterisation of nanoformulations formed by cationic 1,4-dihydopyridine and calix[4]arene compositions
Source: Data Brief. 2022 Feb 23;41:107988. doi: 10.1016/j.dib.2022.107988 (PMC8891969; doi:10.1016/j.dib.2022.107988)
Supplement: Supplementary file 1 [file mmc1.docx]

**Supplementary material for data article:**

**Data for characterisation of nanoformulations formed by cationic 1,4-dihydopyridine and calix[4]arene compositions**

**Authors**

*Martins Rucins^1*^, Roman Rodik^2^, Aiva Plotniece^1^, Nadiia Pikun^1^, Mara Plotniece^3^, Arkadij Sobolev^1^,* Vitaly Kalchenko*^2^, Karlis Pajuste^1*^*

**Affiliations**

*^1^Latvian Institute of Organic Synthesis, Aizkraukles str. 21, LV-1006, Riga, Latvia*

*^2^Institute of Organic Chemistry, National Academy of Science of Ukraine, Murmanska str. 5, 02660 Kiev, Ukraine*

*^3^ Department of Pharmaceutical Chemistry, Faculty of Pharmacy, Riga Stradiņš University, Dzirciema str. 16, LV-1007, Riga, Latvia*

**Corresponding author’s email address and Twitter handle**

*email address:* [*rucins@osi.lv*](mailto:rucins@osi.lv) *(M.R.),* [*kpajuste@osi.lv*](mailto:kpajuste@osi.lv) *(K.P.)*

*Twitter: @A. Plotniece*

Figure S1. DLS size distribution analysis for freshly prepared composition 2/1

Figure S2. DLS size distribution analysis for prepared composition 2/1 after 20 days

Figure S3. DLS size distribution analysis for freshly prepared composition 3/1

Figure S4. DLS size distribution analysis for prepared composition 3/1 after 20 days

Figure S5. DLS size distribution analysis for freshly prepared composition 4/1

Figure S6. DLS size distribution analysis for prepared composition 4/1 after 20 days


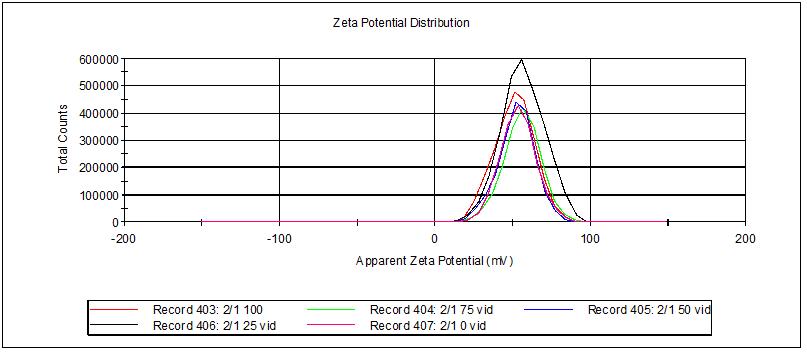


Figure S7. Zeta-potential analysis for freshly prepared composition 2/1


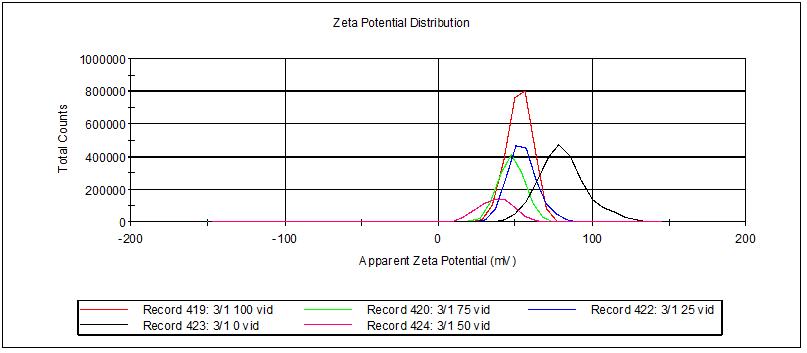


Figure S8. Zeta-potential analysis for freshly prepared composition 3/1


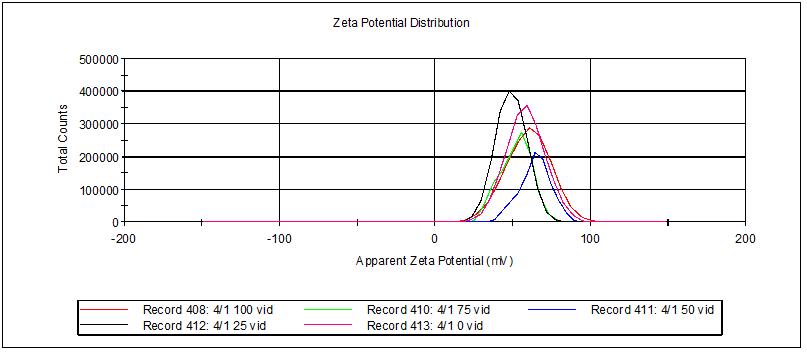


Figure S9. Zeta-potential analysis for freshly prepared composition 4/1


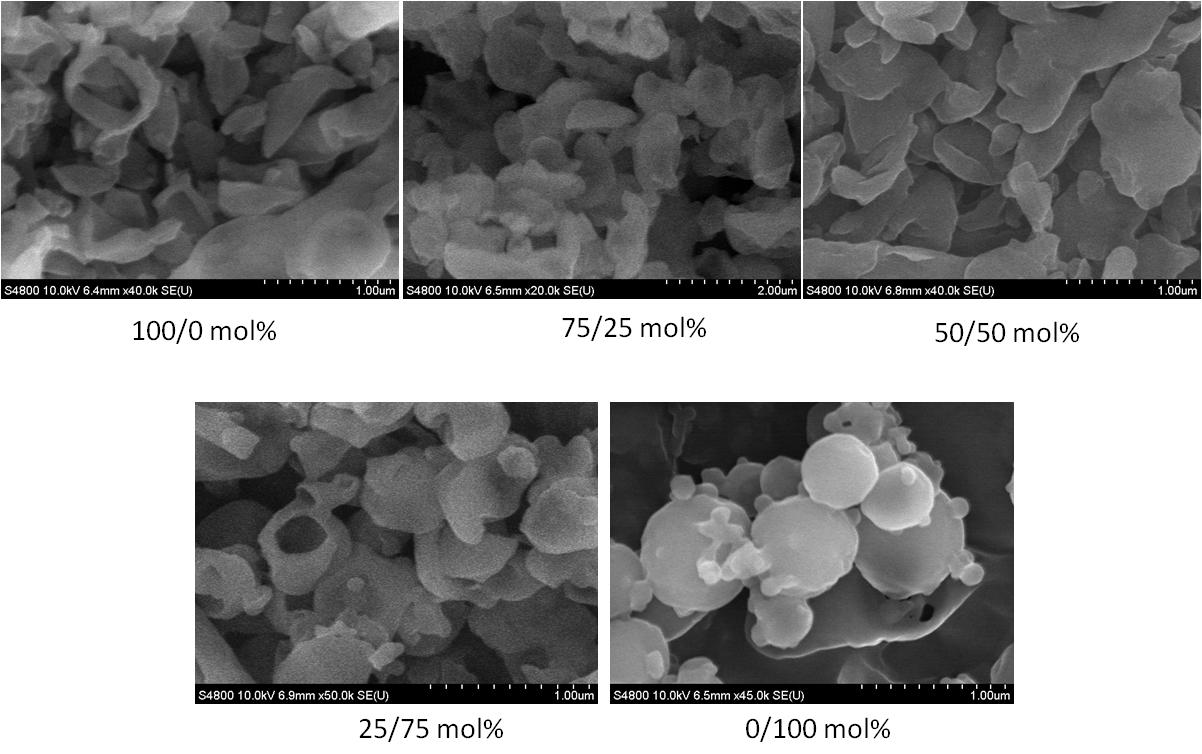


*Figure S10. The SEM images of calix[4]arene* ***2****/1,4-DHP* ***1*** *compositions for freeze-dried samples*
